# Impact of Vertical and Horizontal Skin Incisions on Outcome Measures in Tracheostomies

**DOI:** 10.7759/cureus.54142

**Published:** 2024-02-13

**Authors:** Shaila Sidam, Angam Nasi, Vikas Gupta, Saurabh Saigal, Anjan K Sahoo, Utkal P Mishra, Ganakalyan Behera

**Affiliations:** 1 Otolaryngology - Head and Neck Surgery, All India Institute of Medical Sciences, Bhopal, Bhopal, IND; 2 Anesthesiology and Critical Care, All India Institute of Medical Sciences, Bhopal, Bhopal, IND

**Keywords:** vertical, tracheostomy, incision, horizontal, complication

## Abstract

Abstract

Tracheostomy is a life-saving procedure in which an opening is created in the anterior wall of the trachea. Different skin incision types are administered in tracheostomy procedures, predominantly vertical and horizontal. Various literature on the skin incision types in tracheostomy had contradictory findings, with different studies observing that one skin incision type had better outcomes than its counterpart. Hence the objective of this study was to compare the outcomes associated with vertical and horizontal skin incisions in patients undergoing tracheostomy.

Method

The present study assessed the outcome measures between the two incision types (vertical and horizontal) in tracheostomy. A prospective longitudinal study was done based on an academic tertiary hospital in Bhopal, Madhya Pradesh. Participants above 18 years who underwent tracheostomy were enrolled in the study and followed up over six months during intraoperative, immediate, within seven days, and long-term periods.

Result

In intraoperative complications, bleeding was most common (n = 15, 16.7%), followed by passage of tube into false tract (n = 6, 6.7%) and saturation drop (n = 2, 2.2%). Immediate complications comprised T-tube blockage (n = 4, 4.4%) and bleeding (n = 1, 1.1%). Complications within seven days occurred only in the horizontal group in which stomal site ulceration (n = 4, 6.7%) and delayed bleeding (n = 2, 3.3%) was seen, and one participant had unintended decannulation. In the long term, complications observed were stomal granulation (n = 9, 19.1%), dysphagia (n = 7, 14.9%), and unintended decannulation (n = 4, 8.5%).

Conclusion

In the current study, the most common intraoperative complication was bleeding, the immediate complication was tube dislodgement, and tracheostomy site ulcer was the most common complication within seven days, similar to the literature findings.

## Introduction

The word "tracheostomy" was derived from two Greek words that collectively mean “I cut the trachea” [1] and was first coined by Heister in 1739. Tracheostomy is defined as a procedure of creating an opening in the anterior wall of the trachea, commonly performed on critically sick patients [2]. The first successful tracheostomy was done by Antonio Musa Brassavola in 1546, who used it for relieving airway obstruction due to peri tonsillar abscess [3]; the principles of surgical tracheostomy (ST) were first described by Jackson in 1909. Its use in the Intensive Care Unit (ICU) gained popularity during the polio epidemic in the 1950s [4].

There are four types of tracheostomy: (1) emergency tracheostomy, when the obstruction is complete or almost complete and there is an urgent need to establish the airway; (2) elective tracheostomy, which is planned, often temporary, and closed post-indication; (3) permanent tracheostomy, when the tube is permanently placed; (4) percutaneous dilatational tracheostomy, usually performed in ICU patients when the patient is already intubated and monitored.

There are multiple indications for tracheostomy which are broadly classified, based on timing: (1) emergency tracheostomy for upper airway obstruction; (2) elective tracheostomy for prolonged mechanical ventilation or as a part of another surgical procedure; and (3) in cases of chronic aspiration [5,6]. There are no absolute contraindications to tracheostomy, however, anterior exuberant neck cellulitis could qualify as one. However, in patients with a terminal prognosis, careful consideration must be given to the psychological effect and the quality of life aspects.

Complications can be immediate (hemorrhage, air embolism, and local damage), intermediate (extubation, obstruction, subcutaneous emphysema, infection, and fistula), late (tracheocutaneous fistula, trachea-oesophageal fistula, and tracheal stenosis) [7].

## Materials and methods

A prospective longitudinal study was conducted in an academic tertiary care hospital in Central India. Ethical clearance was obtained from the institutional human ethical committee, and informed consent was obtained from the participants. The study period was from September 2021 to October 2022. A total of 90 participants above the age of 18 years were enrolled in the study, of which 30 participants were in the vertical skin incision group and 60 were in the horizontal skin incision group. Vertical incision was performed in each third case of tracheostomy. Hence, the ratio of vertical and horizontal skin incision types was in the proportion of 1:2. Proper history taking and a detailed examination of the neck and larynx along with investigations such as X-rays and CT scans of the neck and chest were performed. Patients were assessed in detail regarding comorbidities, neurological conditions, and other causes. The procedure was performed by one of the senior authors. While performing the procedure, the following intraoperative complications were assessed: bleeding, the passage of the tube into the false tract, foreign body (cartilage) aspiration, fall in saturation, injury to cricoid/ first tracheal ring/ posterior tracheal wall, and air embolisms.

The participants were followed up over six months post tracheostomy for complications that occurred in the following time frames: immediate, within seven days, and long-term. An evaluation was conducted to assess immediate complications, including subcutaneous emphysema, pneumothorax, pneumomediastinum, T-tube blockage or dislodgement, bleeding, and any other potential complications. In complications within seven days, the patients were assessed for delayed bleeding, hematoma, tracheostomy site ulcer, unintended decannulation, dysphagia, and cellulitis. For assessment of long-term complications factors taken were tracheal stenosis, tracheo-cutaneous fistula, tracheo-esophageal fistula, tracheo-arterial fistula, tracheomalacia, dysphagia, unintended decannulation, and stomal granulation. Data entry and cleaning were done in Microsoft Excel, and data analysis was done using SPSS (IBM Corp., Armonk, NY) and R Studio (R Foundation for Statistical Computing, Vienna, Austria). Numerical variables were summarized as mean and standard deviation if normally distributed or median and interquartile range if non-normally distributed, while categorical variables were summarized as number and percentage. The t-test was used for comparing numerical outcomes, and the chi-square test for categorical outcomes among the two test groups, which were vertical and horizontal skin incision types.

## Results

**Figure 1:**
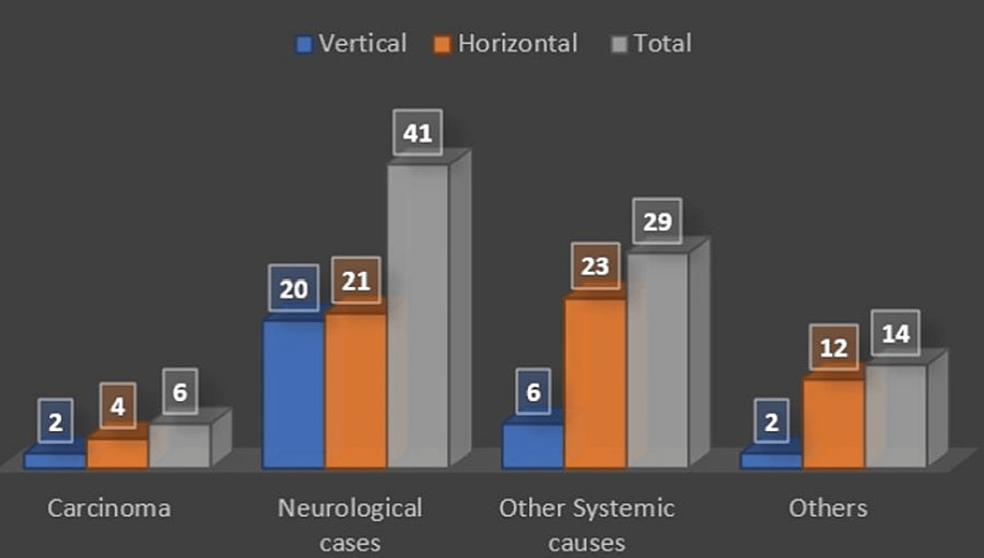
Diagnosis of cases

The patients’ mean age at the time of enrollment was 49.7±17.2 years and the study population predominantly comprised males (n = 62, 68.9%). Half of the participants had comorbidities (n = 45, 50.0%), which included diabetes mellitus, hypertension, and hypothyroidism. The patients were diagnosed with mostly neurological cases (n = 41, 45.6%), followed by other systemic causes like sepsis, disseminated tuberculosis, hepato-renal syndrome, etc. (n = 29, 32.2%), others (n = 14, 15.6%), and carcinoma (n = 6, 6.7%) (Figure 1). 

**Figure 2:**
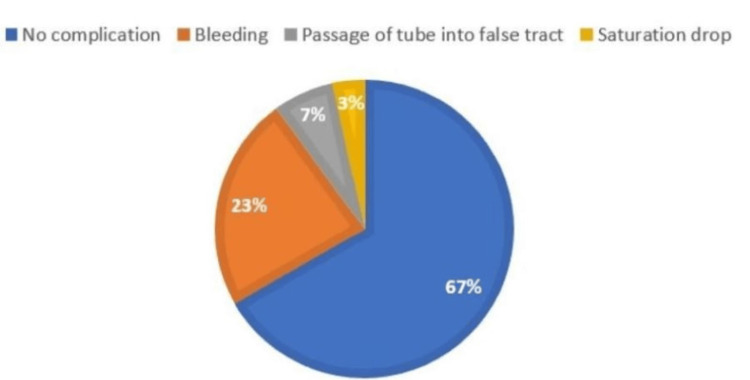
Intraoperative complications (vertical)

**Figure 3:**
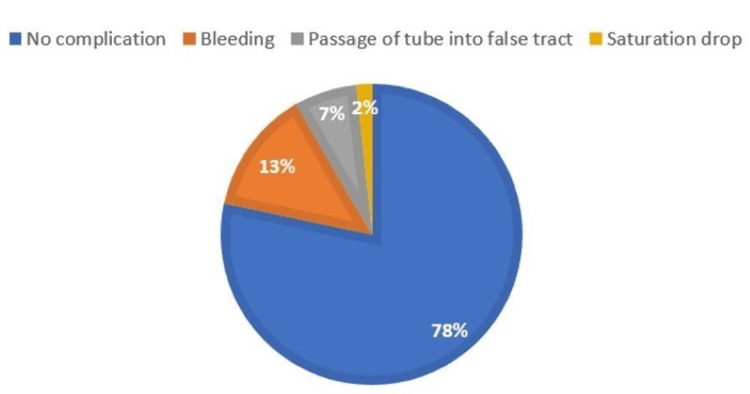
Intraoperative complications (horizontal)

The main indication for tracheostomy was prolonged intubation (n = 83, 92.2%). Patients were grouped according to the skin incision types, i.e., vertical or horizontal skin incisions; 30 vertical (33.3%) and 60 horizontal (66.7%). In intraoperative complications, the most common complication was bleeding (n = 15, 16.7%; vertical=23.3%, horizontal=13.3%), followed by the equal incidence of both vertical and horizontal with the passage of tube into false tract (n = 6, 6.7%), and saturation drop (n = 2, 2.2%; vertical=3.3%, horizontal=1.7%) with p-value = 0.47 among the two groups (Figures 2-3). 

**Figure 4:**
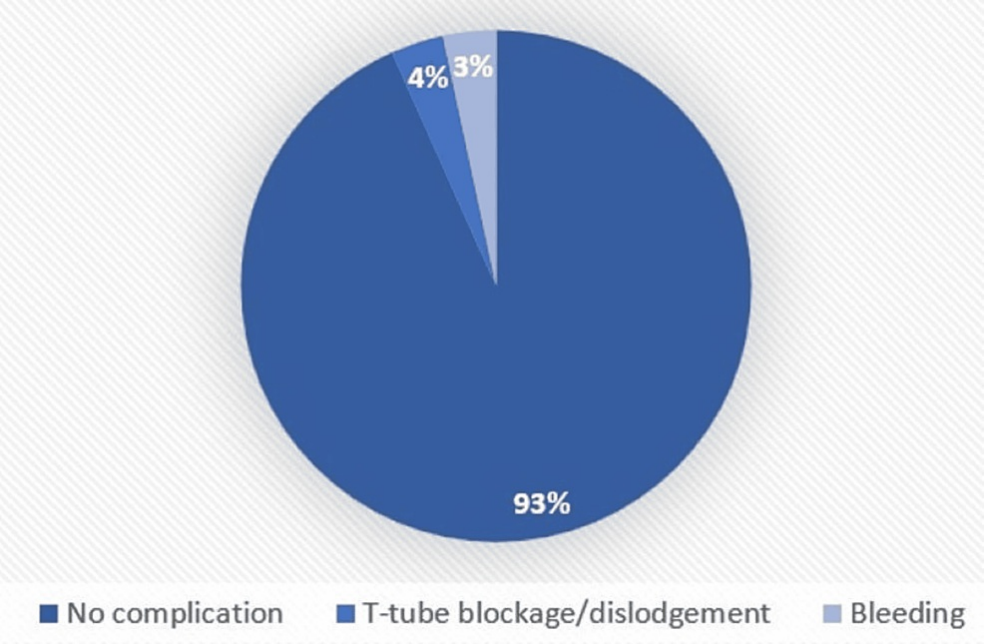
Immediate complications (vertical)

**Figure 5:**
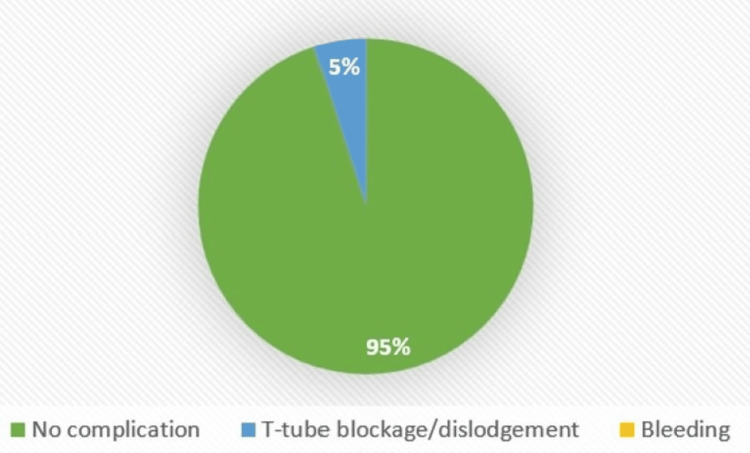
Immediate complications (horizontal)

Regarding immediate complications, only T-tube blockage (n = 4, 4.4%; vertical = 3.3%, horizontal = 5.0%) and bleeding occurred (n = 1, 1.1%, seen only in vertical = 3.3%), with a p-value of 0.53 among the groups (Figures 4-5). 

**Figure 6:**
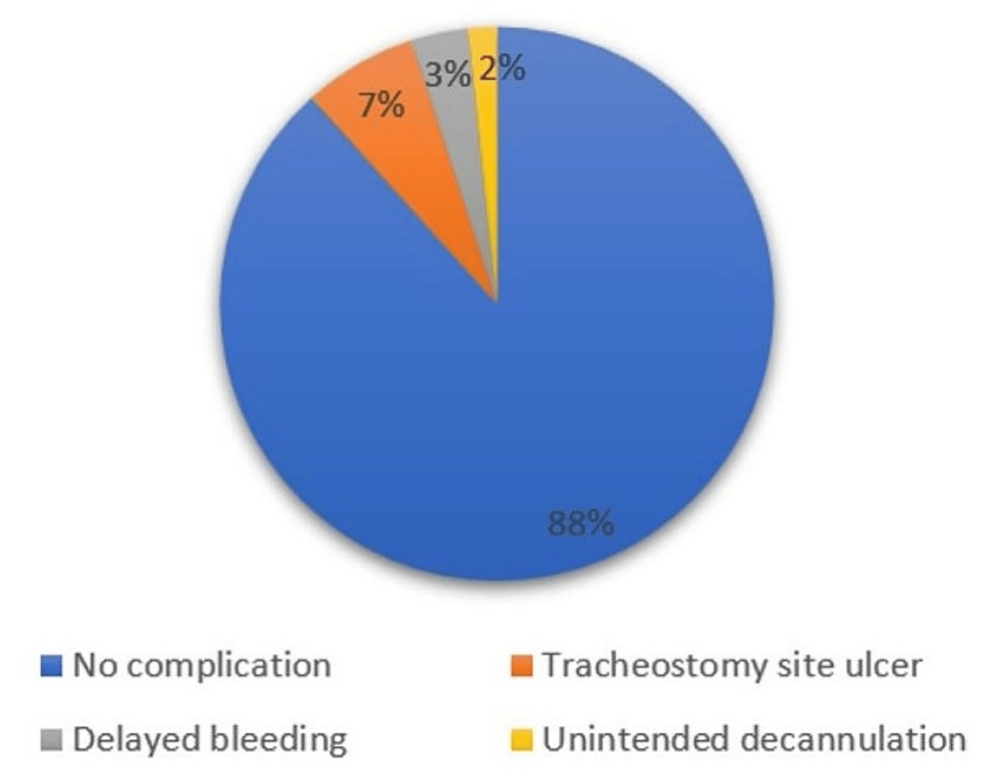
Complications within seven days (horizontal)

**Figure 7:**
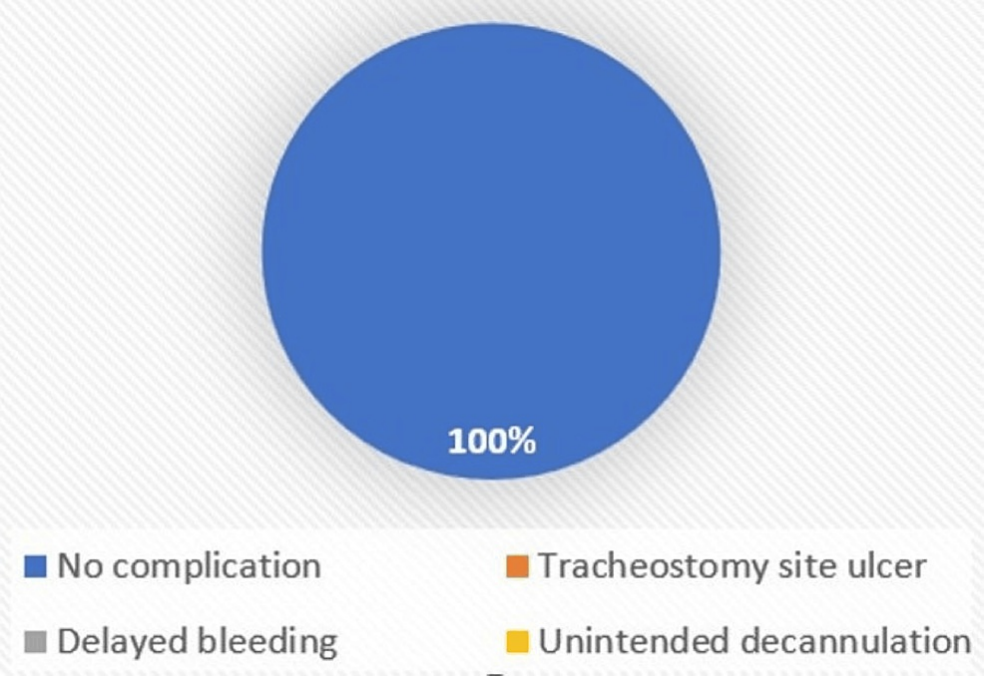
Complications within seven days (vertical)

Complications within seven days were seen only in horizontal skin incision type, comprising tracheostomy site ulcers (n = 4, 6.7%), delayed bleeding (n = 2, 3.3%), and unintended decannulation (n = 1, 1.7%) with p-value = 0.44 (Figures 6-7). 

**Figure 8:**
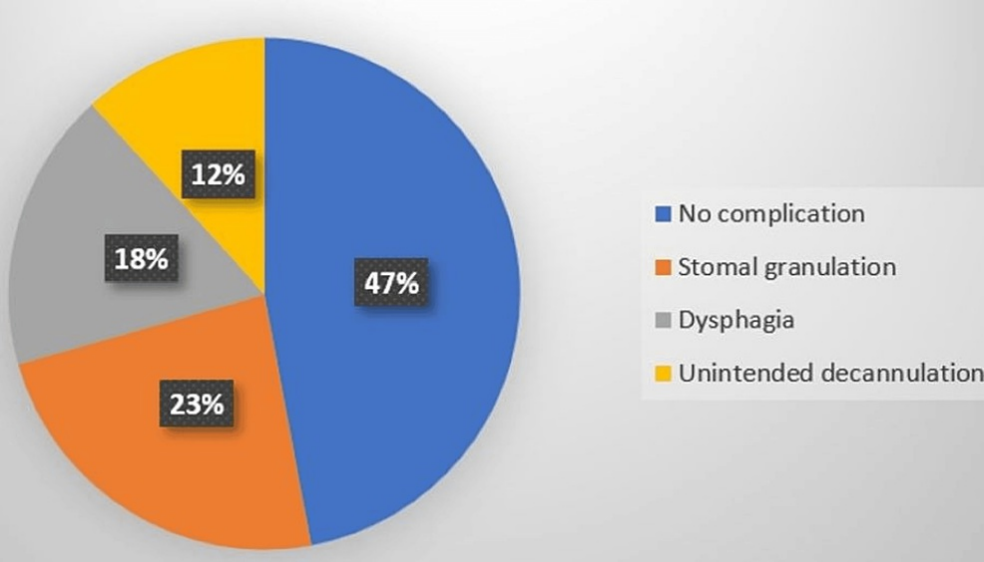
Long-term complications (vertical)

**Figure 9:**
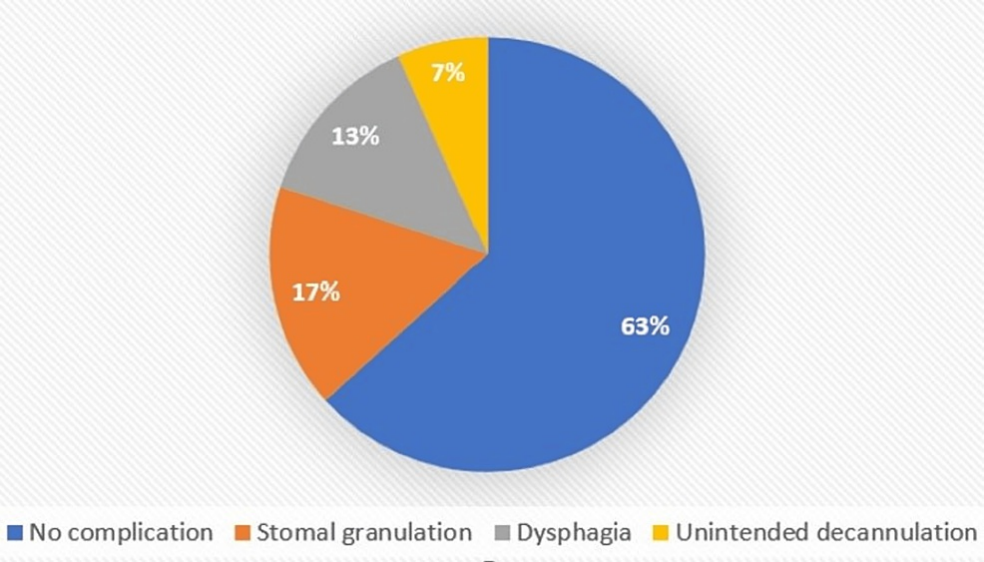
Long-term complications (horizontal)

In the current study, almost half of the participants could not be followed up for long-term complications due to mortality and loss to follow-up, and of the remaining (n=47) stomal granulation (n = 9, 19.1%; vertical = 23.5%, horizontal = 16.7%), followed by dysphagia (n = 7, 14.9%; vertical = 17.6%, horizontal = 13.3%), and unintended decannulation (n = 4, 8.5%; vertical=11.8%, horizontal=6.7%) was observed with a p-value = 0.71 (Figures 8-9).

## Discussion

Tracheostomy is a common surgical procedure performed to relieve the upper airway obstruction. It is performed for ventilation-dependent cases in the intensive care unit or is done in cases with impending upper airway obstruction which can be due to infection, inflammation, facial trauma, or in head and neck cancer patients.

In the current study, 90 participants subjected to vertical and horizontal incisions were assessed on various factors including patient characteristics, complications, and decannulation. The study design for the current study was an observational prospective longitudinal study in which the participants were followed up for a period of six months. The participants were followed up thrice; after the first month, the third month, and the sixth month. This is in line with a study by Menon et al. who had also followed up for a period of six months for the tracheostomy patients [8]. The study setting was a single-centre study, conducted in an academic tertiary care hospital at AIIMS Bhopal. This setting was chosen for convenience as per the training of the resident. The majority of studies were also single-center studies (Wahla et al. [9], Bathula et al. [10], Prasad et al.) [11]. However, there are few other studies that were multi-center in design (Pablo et al. [12], Hansson et al. [13]). 

The study population included patients who were above 18 years of age with no history of prior tracheostomy, and procedures done only by ENT and Anaesthesia department. The age criteria were taken as above 18 years as for pediatric patients there is a variation from adults in terms of various factors including anatomy, techniques, indications, complications, and decannulation in line with other studies including systematic reviews by Singh et al. [14] and Ferro et al. [15].

In the present study, the most common intraoperative complication was bleeding (n = 15, 16.7%) followed by passage of the tube into the false tract (n = 6, 6.7%) but there was no statistical significance noted between the two incision types. A systematic review and meta-analysis by Delaney et al. also found the incidence of bleeding to be the most common complication [16]. It was however observed to be higher in vertical incisions (n = 7, 23.3%) as compared to horizontal (n = 8, 13.3%), which could be due to the smaller sample size in the vertical group or due to the surgeon’s skill and expertise.

In immediate complications, the complication seen the most in the present study was tube dislodgement among four patients (V=1, H=3), similar to findings by Glossop et al. [17], and Pal et al. [18] This could be due to the procedure being done in bedridden, elderly patients who were unable to take care of the tracheostomy tube by themselves or due to the non-sutured neck tie to skin [19].

The current study assessing complications within seven days found tracheostomy site ulcers (n = 4, 4.5%) to be the most common complication in this period. This is similar to the findings of a study by Lim et al. on skin incision types in percutaneous tracheostomy [20] and Gummusoy et al. [21].

Long-term complications observed were stomal granulation (n = 9, 19.1%), dysphagia (n = 7, 14.9%), and unintended decannulation (n = 4, 8.5%) in the vertical skin incision type as compared to its horizontal counterpart (36.7% i.e., about 1/3rd) but there was no statistical difference noted. In a study by Ahmed et al., which was done with a study population of 534 patients, they observed only two patients had accidental or unintended decannulation, which is also similar to the findings of the current study [22]. There are various factors that may lead to unintended decannulation like poor sensorium (bedridden patients unable to take care of themselves), poorly secured T-tube, excessive cough, obesity, and short neck. No life-threatening complications such as tracheal stenosis, tracheo-esophageal fistula, tracheo-arterial fistula, or tracheomalacia were seen in the present study.

Certain assessment criteria were taken for decannulation which included level of consciousness, cough effectiveness, secretions, swallowing, capping tolerability, and mobility of vocal cords. This is in line with other studies which include Enrichi et al. [23], and Bishnoi et al. [24] who had also taken similar assessment criteria for decannulation. In the current study, there were no significant differences in the predictors of decannulation based on the vertical and horizontal skin incision types. Also, there were no studies found on decannulation predictors based on skin incision type in surgical tracheostomy.

In the present study, 46% of patients were decannulated successfully, which is parallel to the findings in various studies including a study by Park et al. [25] in which it was seen that 43% of patients were decannulated successfully. Another study by Kim et al. also observed successful decannulation in about 40% of patients [26].

The limitation of the current study was that it was single-center in design and had a small sample size. Another limitation was the population of the study, critically ill patients, most of whom were admitted to the ICU, resulting in a high mortality rate overall.

## Conclusions

There are limited studies on the impact of the outcomes relating to different skin incision types in tracheostomy. Vertical incision is commonly used for emergency tracheostomy and horizontal incision for elective tracheostomy. This study did not find any significant difference among both incision types in terms of the outcome of tracheostomy. Common complications noted were bleeding, unintentional tube decannulation, and tracheostomy site ulceration. These findings provide important clinical data and could lead to changes in the practice regarding the type of incision used for tracheostomy.
